# Changes in the Oxidation-Reduction State of Human Dermal Fibroblasts as an Effect of Lomefloxacin Phototoxic Action

**DOI:** 10.3390/cells11121971

**Published:** 2022-06-19

**Authors:** Justyna Kowalska, Klaudia Banach, Zuzanna Rzepka, Jakub Rok, Marta Karkoszka, Dorota Wrześniok

**Affiliations:** Department of Pharmaceutical Chemistry, Faculty of Pharmaceutical Sciences in Sosnowiec, Medical University of Silesia in Katowice, Jagiellońska 4, 41-200 Sosnowiec, Poland; jkowalska@sum.edu.pl (J.K.); kbanach@sum.edu.pl (K.B.); zrzepka@sum.edu.pl (Z.R.); jrok@sum.edu.pl (J.R.); d200971@365.sum.edu.pl (M.K.)

**Keywords:** fluoroquinolones, phototoxicity, oxidative stress, lomefloxacin

## Abstract

Phototoxicity induced by antibiotics is a real problem in health care. The discontinuation of antibiotic therapy due to a phototoxic reaction can lead to the development of resistant strains. Fluoroquinolones are widely used antibiotics that exhibit phototoxic activity under UVA radiation. The purpose of the study was to examine the redox status of human dermal fibroblasts exposed to UVA radiation and treated with lomefloxacin, the most phototoxic fluoroquinolone. Lomefloxacin alone was found to have an antiproliferative activity on fibroblasts by affecting the cell cycle. In addition, the drug caused a redox imbalance associated with the decreased expression of catalase and glutathione peroxidase. UVA radiation increased the drug cytotoxicity and oxidative stress induced by lomefloxacin. The decrease in cell viability was accompanied by a high level of reactive oxygen species and extensive changes in the antioxidant levels. The revealed data indicate that the phototoxic action of lomefloxacin results from both increased reactive oxygen species production and an impaired antioxidant defense system. Considering all of the findings, it can be concluded that lomefloxacin-induced phototoxic reactions are caused by an oxidoreductive imbalance in skin cells.

## 1. Introduction

The adverse cutaneous reactions to drugs are a serious problem in health care. They occur in an estimated 10% of hospitalized patients [1]. One of the cutaneous adverse reaction types is photosensitivity. It is important to note that drug-induced photosensitivity is often underdiagnosed. This aspect is particularly related to antibiotic therapy, in which patients prefer to stop the drug if they suffer sunburn-like reactions rather than consult a doctor for a formal diagnosis [2]. A shortened duration of antibiotic administration may contribute to the development of bacterial antimicrobial resistance, one of the most serious threats to global health [3].

Drug-induced photosensitivity is defined as a cutaneous adverse event resulting from a combination of photoactive drug administration and sunlight exposure. Photosensitivity reactions are induced by specific regions of the electromagnetic spectrum. UVA radiation is considered to be the primary trigger of photosensitivity events because it penetrates deeply into the skin reaching the dermis [4,5]. UVA radiation, which is in the 320 nm to 380 nm range, represents about 95% of the entire UV radiation reaching the Earth’s surface. Both the UVA/UVB ratio and UV irradiance are determined by various factors, such as altitude, time of day, latitude, and season. In addition, there are many artificial sources of UVA radiation, such as sunbeds, lamps intended for phototherapy, or photocuring [6]. Photoactive drugs are able to absorb normally innocuous levels of radiation, creating harmful photoproducts. More than 300 drugs are known to be photosensitizers and include many of the commonly used drugs such as nonsteroidal anti-inflammatory drugs, cardiovascular drugs, antibiotics, antidiabetic drugs, antipsychotics, antidepressants, and antihistamines [7,8]. Drug-induced photosensitivity clinically manifests as exaggerated sunburn, hyperpigmentation, erythematous/ edematous/ blistering lesions, photoonycholysis, as well as telangiectasia [9].

Fluoroquinolones (FQs), which are antibiotics used in the therapy of a wide range of bacterial infections, are a group of drugs considered to be associated with photosensitivity reactions. FQs cause two types of photosensitivity reactions: phototoxicity and photoallergy, and phototoxic reactions are more common [10]. The severity and incidence of phototoxic reactions differ among FQs, and lomefloxacin (LF) has been reported to have the greatest phototoxicity potential [11,12,13]. The phototoxic action of LF is attributed to its susceptibility to UVA-induced photolysis. The presence of a fluorine atom at position 8 enhances UV-stimulated chemical modifications of LF, resulting in the decomposition of the drug molecule, and the generation of reactive carbene and reactive oxygen species, which are extremely harmful to nearby cells [14,15,16]. We previously demonstrated that LF binds to melanin and therefore may accumulate in pigmented tissues such as skin, thus possibly contributing to dermal side effects of the drug [17,18]. The analysis of phototoxicity of LF to melanocytes (melanin-producing cells) allowed us to demonstrate the induction of oxidative stress as well as changes in melanogenesis and cell viability [19,20].

It is important to note that the skin is a multilayered organ whose physiology depends on a complex interaction of different cell types. Moreover, the skin’s neuroendocrine system, which consists of cells in the epidermis and dermis, as well as nerve endings and sensory receptors and immune cells, is responsible for the interaction between internal organs and the environment [21]. A key role in skin homeostasis is played by fibroblasts. They are the most prevalent cells in the dermis. The distinctive feature of fibroblasts is their ability to synthesize, remodel and deposit collagen in the non-collagen extracellular matrix, affecting skin firmness and thickness. Fibroblasts interact with each other and with neighboring cells through the production and release of a wide range of cytokines and growth factors, affecting various aspects of skin physiology [22,23,24,25]. An important external factor affecting skin homeostasis is UV radiation. The electromechanical energy of radiation is transformed into immunological, hormonal, and nervous signals regulating skin and body homeostasis. On the other hand, the absorption of UV radiation by biologically relevant molecules may contribute to pathophysiological processes [21].

Considering that the pathomechanism of phototoxicity is complex and involves different types of skin cells, we decided to analyze the cyto- and phototoxicity of LF to human dermal fibroblasts. It allowed us to compare the response of pigmented and non-pigmented cells to the phototoxic action of this drug.

## 2. Materials and Methods

### 2.1. Chemicals and Reagents

Lomefloxacin hydrochloride, penicillin G, amphotericin B, H2DCFDA (2′,7′-dichlorofluorescein diacetate), SIGMAFAST™ Protease Inhibitor Cocktail Tablet, and Phosphatase Inhibitor Cocktail 3, Dulbecco’s phosphate-buffered saline (DPBS) with MgCl_2_ and CaCl_2_, phosphate buffered saline (PBS),and Fibroblast Growth Medium were obtained from Sigma Aldrich Inc. (St. Louis, MO, USA). A Pierce BCA Protein Assay Kit, ECL Western Blotting Substrate, and Hoechst 33342, CellROX™ Green Reagent were obtained from Thermo Fisher Scientific (Waltham, MA, USA). GAPDH (14C10) Rabbit mAb, SOD1 (71G8) Mouse mAb, SOD2 (D9V9C) Rabbit mAb, Catalase (D4P7B) Rabbit mAb, and GPx1 (C8C4) Rabbit mAb were obtained from Cell Signaling (Danvers, MA, USA), and Anti-Rabbit IgG (A154), Anti-Mouse IgG, Tween-20, RIPABuffer and PVDF membranes were obtained from Sigma-Aldrich Inc. (St. Louis, MO, USA). Neomycin sulfate was obtained from Amara (Kraków, Poland). Trypsin/EDTA solution was purchased from Cascade Biologics/Gibco (Carlsbad, CA, USA). Solution 3 (1 μg/ mL DAPI, 0.1% triton X-100 in PBS), Solution 5 (VB-48TM, propidium iodide-PI, acridine orange—AO), NC-Slide A8 and Via-1-Cassette (AO and DAPI fluorophores) were obtained from ChemoMetec (Lillerød, Denmark). Cell Proliferation Reagent WST-1 was produced by Roche GmbH (Mannheim, Germany). Other chemicals were from POCH S.A. (Gliwice, Poland).

### 2.2. Cell Culture and the Exposure to Lomefloxacin and UVA Radiation

Fibroblasts were acquired from Sigma Aldrich Inc. (St. Louis, MO, USA). The cell line was cultured in an all-in-one ready-to-use fibroblast growth medium. The treatment with lomefloxacin started 48 h after seeding for cells. The medium was then removed and the cells were treated with a drug for 24 h. After exchanging the medium or drug solutions with DPBS, cells were irradiated with UVA for 30 min (1.3 J/cm^2^) at an intensity of 720 µW/cm^2^ using a lamp BVL-8.LM (VilberLourmat, Collégien, France). The lamp emits UVA radiation of 365 nm wavelength. The dose of UVA radiation applied was chosen on the basis of preliminary studies [data not published] and was equal to the dose applied in the studies on the analysis of fluoroquinolones phototoxicity on melanocytes [19,20]. Afterwards, DPBS was replaced by the culture medium in all samples, and fibroblasts were cultured for the next 24 h.

### 2.3. Cell Viability Assay

The colorimetric WST-1 kit was purchased from Roche GmbH (Mannheim, Germany). In the WST-1 assay, the amount of formazan dye formed is directly related to the metabolic activity of cells. In brief, fibroblasts were seeded in transparent 96-well plates and exposed the day after to different concentrations of lomefloxacin (range from 0.001 to 1.00 mM) for 24 h. Next, fibroblasts were irradiated in DPBS. Cells were then incubated in a fresh medium for the next 21 h. Afterward, 10 µL of WST-1 were added to 100 µL of culture medium in each well, and the incubation was continued for another 3 h. Absorbance was measured at 450 nm (690 nm-reference wavelength) in an Infinite 200 PRO (TECAN, Männedorf, Switzerland) microplate reader. The controls were normalized to 100% and results were expressed as the percentage of the controls.

### 2.4. Cell Proliferation Assay

Cell proliferation was evaluated using a fluorescent imaging cytometer. The analysis was based on the staining of non-fixed cells with acridine orange (total cells population) and DAPI (dead cells). Fibroblasts were seeded in a Petri dish and incubated for 48 h. Cells were then treated with lomefloxacin (0.5 mM) for 24 h. Next, the cells were exposed to UVA radiation. After 24 h, cells were trypsinized and then counted using a fluorescent imaging cytometer NucleoCounterNC-3000 (ChemoMetec, Lillerød, Denmark). In this method, non-fixed cells are loaded into Via1-Cassettes (ChemoMetec, Lillerød, Denmark) and the number of viable cells was determined.

### 2.5. Cell Cycle Analysis

Fixed cell cycle-DAPI assay cell cycle analysis by DNA content measurement was analyzed by the use of a NucleoCounter NC-3000 (ChemoMetec, Lillerød, Denmark)-fluorescence image cytometer. The fibroblasts were incubated with lomefloxacin at a concentration of 0.5 mM for 24 h. After incubation, the cells were irradiated and then incubated in the medium. Subsequently, the fibroblasts were trypsinized and fixed in ethanol (70%). After 48 h, alcohol was removed and cells were washed with PBS. The cells were then stained with Solution 3 according to the producer’s protocol. Based on the results, the relative ratios of G1/S and G2-M/S were calculated.

### 2.6. H2DCFDA Assay

DCFDA is a cell-permeable molecule that is hydrolyzed to the 2′,7′-dichlorodihydrofluorescein carboxylate anion form in the cytosol by intracellular esterases, and then oxidized by intracellular reactive oxygen species to form the fluorescent compound, dichlorofluorescein. The fluorescence intensity (λex = 485 nm, λem = 530 nm) was measured using the microplate reader Infinite 200 Pro (TECAN, Männedorf, Switzerland). Fibroblasts (5000 cells per well) were added to dark, clear bottom 96-well microplates according to DCFDA kit protocol and incubated at 37 °C in 5% CO_2_ for 48 h to allow adherence. Then the drug, at a concentration of 0.5 mM, was added to the cells, and in the next step, fibroblasts were irradiated. Twenty-four hours after irradiation, the cells were treated with H2DCFDA (concentration: 20 μM) in the dark for 30 min and washed twice with PBS. The fluorescence was measured and the results obtained were expressed as a percentage of the control cells.

### 2.7. Confocal Microscopy Imaging

Oxidative stress analysis and cell morphology were conducted by using the laser confocal microscope Nikon Eclipse Ti-E A1R-Si controlled by Nikon NIS Elements AR software (Nikon Instruments, Amsterdam, The Netherlands). Fibroblasts were cultured in 4-well chamber slides. After exposure to LF and UVA radiation, according to the procedure presented in Section 2.2, cells were incubated with CellROX^®^ Green Reagent (5 μM, Thermo Fisher Scientific, Waltham, MA, USA) and Hoechst 33342 (5 μg/mL, Thermo Fisher Scientific, Waltham, MA, USA) for 30 min., washed thrice in PBS, and imaged. Hoechst 33342 stains nuclei and CellROX^®^ dye were used for detecting oxidative stress. CellROX^®^ Green Reagent in the oxidized form is a bright green fluorescent, while in the reduced state it exhibits very weak fluorescence. The confocal imaging in transmitted light was applied to estimate the morphology of the cells.

### 2.8. Western Blotting Analysis

After the treatment and irradiation, fibroblasts were lysed using an RIPA buffer containing phosphatase and protease inhibitors. Lysates were centrifuged and stored AT −86 °C. Next, the protein concentrations in the supernatants were determined spectrophotometrically using a Pierce™BCA Protein Assay Kit (Waltham, MA, USA) according to the producer’s protocol. Protein extracts (20 μg/lane) were separated on a 10% SDS-polyacrylamide gel electrophoresis and transferred to PVDF membranes. Membranes were then incubated for 1 h in a blocking buffer (5% non-fat milk in TBST - Tris-buffered saline with Tween 20). The analyzed membranes were incubated with the primary antibodies: mouse anti-SOD1 (1:1000), rabbit anti-SOD2 (1:1000), rabbit anti-CAT (1:500), rabbit anti-GPx1(1:1000), and rabbit anti-GAPDH (1:1000). Finally, they were washed with TBST and submitted to ECL reagent (Waltham, MA, USA). The analysis was made using a G:Box Chemi-XT4 Imaging System and GeneTools Software (Syngene, Cambridge, UK).

### 2.9. The Estimation of Cellular Reduced Glutathione Level

The level of reduced glutathione in fibroblasts was assessed by the use of the NucleoCounter NC-3000 (ChemoMetec, Lillerød, Denmark) fluorescence image cytometer. Cell vitality was assessed using Solution 5 reagent (VB-48TM, propidium iodide, and acridine orange). VitaBright-48™ is a fluorescent dye emitting fluorescence when bound to reduced thiols (GSH) [26]. The cells were seeded in Petri dishes and after 48 h fibroblasts were exposed to the lomefloxacin at a concentration of 0.5 mM. The medium was then removed and the cells were exposed to UVA radiation. After 24-h incubation in the medium, the cells were harvested by trypsinization and counted (the cytometric technique). In an experiment aimed at analyzing the effect of vitamin C on changes in cell viability induced by lomefloxacin and UVA radiation, cells were post-treated (after irradiation process for 24 h in growth medium) with ascorbic acid at a concentration of 1 μg/mL (non-toxic vitamin C concentration). The stained cells were applied to an NC-Slide A8, measured using the Vitality assay protocol.

### 2.10. Statistical Analysis

Data were analyzed using GraphPad Prism 8 (GraphPad Software, San Diego, CA, USA). The means and standard deviations of at least three separate experiments conducted in triplicate were calculated. Statistical differences were determined by Student’s *t*-test or two-way ANOVA followed by Tukey’s post-hoc test, as appropriate. In all cases, statistical significance was considered with *p* < 0.05.

## 3. Results

### 3.1. UVA Radiation Induces Cytotoxicity of Lomefloxacin

**Figure 1:**
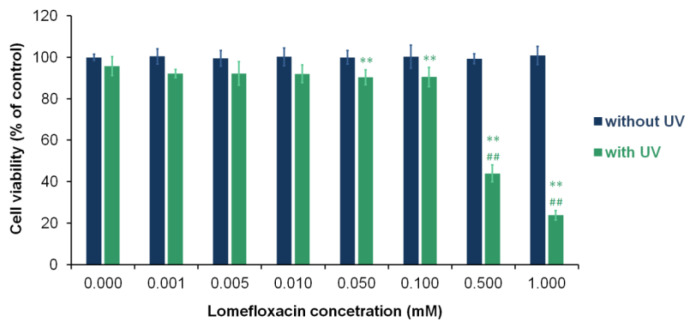
Lomefloxacin decreases the viability of fibroblasts under UVA radiation. Cells were exposed to increasing concentrations of lomefloxacin and UVA radiation at a dose of 1.3 J/cm^2^. The viability of cells was examined by the WST-1 assay. Data are expressed as a percentage of the controls. ** *p* < 0.01 vs. control; ## *p* < 0.01 vs. non-treated cells exposed to UVA radiation.

The influence of LF and UVA radiation on the cell viability of fibroblasts was evaluated by WST-1 assay. In order to assess the cytotoxic effect of the tested drug, cells were treated with LF for 24 h in the concentration range of 0.001–1.00 mM. Afterward, cells were exposed to UVA radiation. As shown in Figure 1 LF, this alone did not reduce the viability of the fibroblasts. The exposure of cells to the drug in concentrations of 0.05–1.00 mM and UVA radiation significantly decreased the viability of fibroblasts by about 10–76%. The highest phototoxic effect was observed in irradiated samples treated with the drug at a concentration of 0.50 mM and 1.00 mM.

Taking into account the noted phototoxic action of lomefloxacin, an analysis of the effect of the drug on fibroblast proliferation and oxidoreductive balance was performed using lomefloxacin at a concentration of 0.5 mM.

### 3.2. Lomefloxacin Suppresses the Proliferation of Fibroblasts

**Figure 2:**
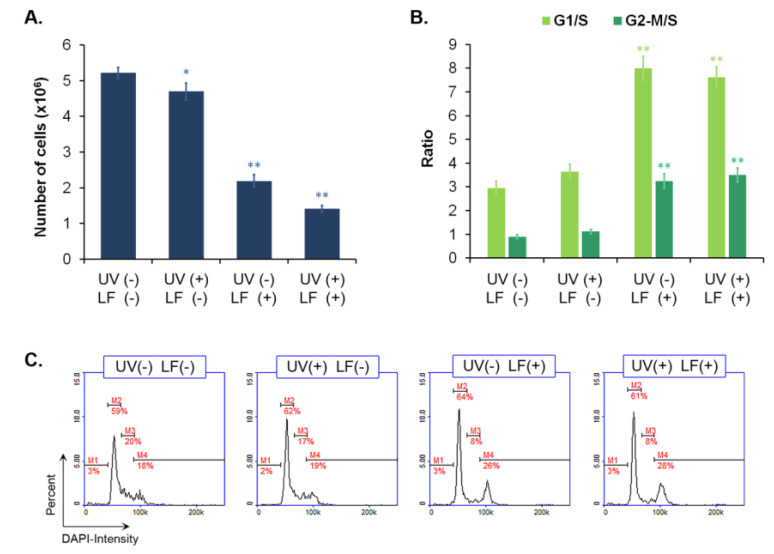
Lomefloxacin alone and in addition to UVA radiation causes the inhibition of fibroblast proliferation affecting the cell cycle. Cell number (**A**) and cell cycle (**B,C**) analyses were performed using lomefloxacin (LF) in the concentration of 0.5 mM and UVA radiation at a dose of 1.3 J/cm^2^. The presented histograms are representative of three independent experiments. Marked cell cycle phases: M1–sub-G1 phase; M2–G1/G0 phase; M3–S-phase; M4–G2/M phase. * *p* < 0.05, ** *p* < 0.01 vs. control.

The estimation of cell proliferation and the cell cycle was conducted using the fluorescence image cytometer NucleoCounterNC-300 (Figure 2). It was observed that UVA radiation did not affect the cell cycle of fibroblasts; however, it did decrease the cell numbers. A stronger antiproliferative effect was demonstrated in the cells exposed to the drug alone and in the samples treated with the drug and UVA. Obtained results showed that LF decreased the cell numbers in treated cultures by about half and increased the relative ratios of G1/S and G2-M/S by over about 5.1 and 2.3, as compared to the control sample. It was also noticed that the drug in combination with UVA irradiation caused a decrease in cell number by a factor of about four and an increase in G1/S and G2-M/S ratios–4.7 and 2.6, respectively, relative to the control. LF-induced cell cycle modifications were mainly due to a decrease in the number of cells in S phase (Figure 2C). This effect was not enhanced by UVA radiation.

### 3.3. UVA Radiation Enhances Oxidative Stress Induced by Lomefloxacin

**Figure 3:**
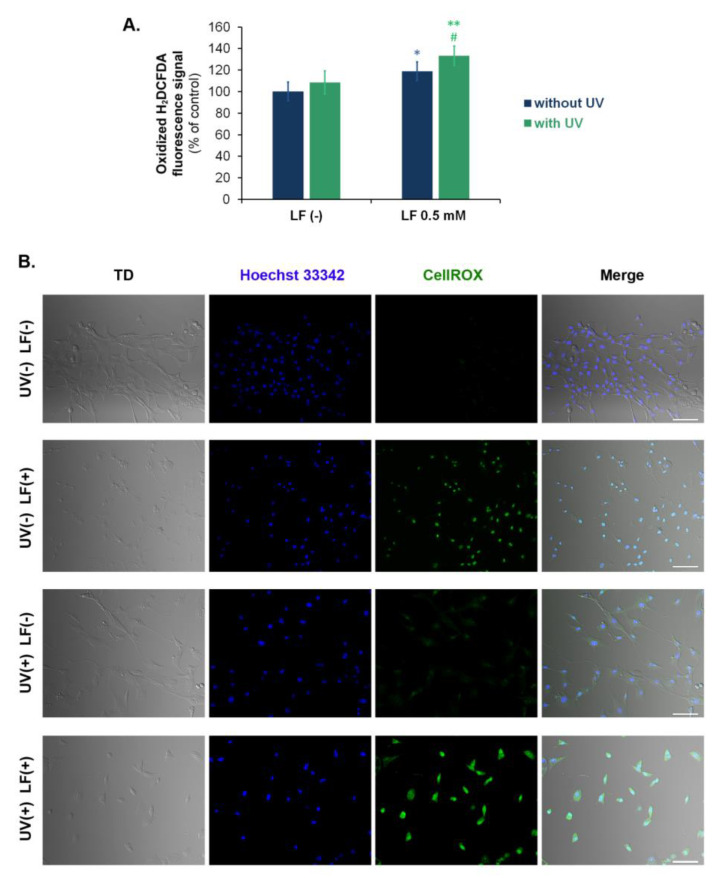
UVA radiation augments lomefloxacin-induced oxidative stress. Cells were exposed to lomefloxacin (LF) at a concentration of 0.5 mM and UVA radiation at a dose of 1.3 J/cm^2^. (**A**). Intracellular level of reactive oxygen species determined by H2DCFDA staining. Data are expressed as % of the controls. * *p* < 0.05, ** *p* < 0.01 vs. control; # *p* < 0.05 vs. non-treated cells exposed to UVA radiation. (**B**). Representative confocal microscopic images of fibroblasts stained with CellROX^®^ Green Reagent for oxidative stress detection (green channel) and Hoechst 33342 for nuclei visualization (blue channel). Cell morphology was evaluated by observations using a transmitted light detector (TD). Scale bar = 100 µm.

H2DCFDA staining was used to detect ROS generation in fibroblasts exposed to LF and UVA radiation. The treatment of cells with LF led to ROS overproduction, but the simultaneous use of the drug and UVA enhanced this effect, an increase of 19% and 38%, respectively, relative to the control (Figure 3A). The confocal microscopic analysis also indicated that LF alone and in addition to UVA radiation caused oxidative stress in cells, whereas irradiated cells treated with LF showed the greater intensity of CellRox fluorescence than the drug-treated fibroblasts (Figure 3B). 

By using confocal imaging in transmitted light, we assessed the cell morphology. It was observed that UVA radiation caused minor alterations in fibroblast morphology, whereas LF alone and in combination with radiation highly changed the shape of the cells. Fibroblasts treated with LF had significantly modified morphology compared to control cells. In the case of cells exposed to LF and radiation, this effect was even more pronounced, because fibroblasts lost the typical shape of the cell line.

### 3.4. The Impact of Lomefloxacin and UVA Radiation on Antioxidant Enzymes Expression

**Figure 4:**
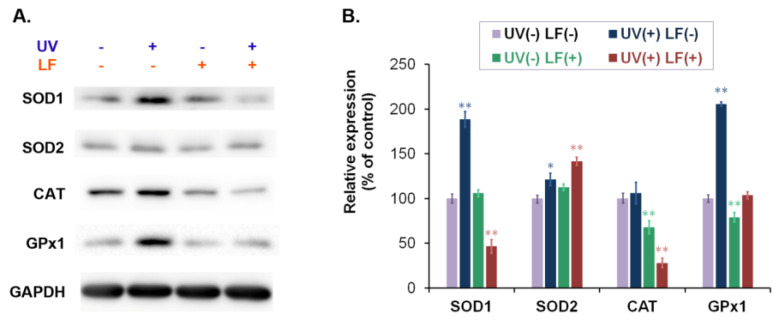
Lomefloxacin alone as well as with UVA radiation modifies the expression of antioxidant enzymes. Cells were exposed to lomefloxacin (LF) at a concentration of 0.5 mM and UVA radiation at a dose of 1.3 J/cm^2^. The levels of superoxide dismutase isoforms 1 and 2 (SOD1, SOD2), catalase (CAT) as well as glutathione peroxidase isoform 1 (GPx1) were examined by western blot analysis. The results are presented as (**A**) representative blot images and (**B**) bar graphs. * *p* < 0.05, ** *p* < 0.01 vs. control.

The levels of antioxidant enzymes in fibroblasts were determined by western blot analysis. We assessed the expression of superoxide dismutase isoforms 1 and 2 (SOD1, SOD2), catalase (CAT), as well as glutathione peroxidase isoform 1 (GPx1). The obtained results are presented in Figure 4 as representative blot images and bar graphs. It was found that UV caused a significant increase in SOD1, SOD2, and GPx1 levels in fibroblasts. LF alone decreased CAT and GPx1 levels by about 32% and 21%, respectively, when compared to the control. The changes in SOD1, SOD2, and CAT expression were also noticed for the combination of UV and LF. The simultaneous exposure of fibroblasts to UVA and drugs in a concentration of 0.5 mM resulted in a decrease in SOD1 and CAT levels of about 53% and 72%, respectively, when compared with the control. Moreover, these two factors (drug and UVA) increased the SOD2 level by about 42%.

### 3.5. Lomefloxacin and UVA Radiation Decrease Vitality of Fibroblasts

**Figure 5:**
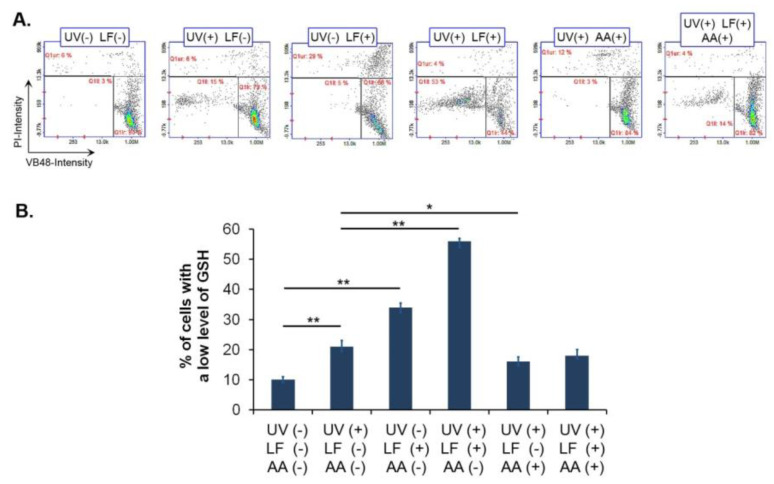
(**A**,**B**) Lomefloxacin alone as well as plus UVA radiation decreases the level of reduced glutathione in fibroblasts. Ascorbic acid (AA) reverses the phototoxic action of lomefloxacin. Cells were exposed to lomefloxacin (LF) at a concentration of 0.5 mM and UVA radiation at a dose of 1.3 J/cm^2^. The presented histograms are representative of three independent experiments. Q1ur and Q1ll-cells with a low level of reduced glutathione; Q1lr-cells with a high level of reduced glutathione. * *p* < 0.05; ** *p* < 0.01.

We analyzed the cellular reduced glutathione level in LF-treated and UVA irradiated fibroblasts (Figure 5). The incubation with the drug caused the increase in the percentage of cells with low reduced glutathione level to about 34%. In the sample exposed to the drug and UVA, 56% of cells exhibited a depletion of reduced glutathione. The obtained results showed that the post-incubation of fibroblasts with vitamin C at a concentration of 1 μg/mL reversed the impact of UVA-activated LF on cell vitality. The number of cells with low reduced glutathione levels decreased by about 40% when vitamin C was applied after the drug and UVA exposure.

## 4. Discussion

The skin, the largest organ of the body, is the site of a variety of oxidative reactions, which are neutralized by endogenous or exogenous antioxidative systems. There is a pool of protective antioxidants in the skin, whose role is to maintain redox homeostasis. Skin exposure to a variety of irritants, such as ionizing and UV radiation, drugs, cosmetic products, and food preservatives, contributes to the increased production of free radicals, which causes a redox imbalance [27]. Insufficient removal of reactive species results in altered cellular metabolism, oxidative damage to biomolecules (lipids, proteins, DNA), cell cycle, and signal transduction dysregulations. Impaired free radical neutralization is associated with many cutaneous disorders ranging from photosensitivity to neoplasia [28].

Skin cells differ in their antioxidant potential and in the range of oxidants to which they are exposed. The melanocytes present in the epidermis are exposed to UVA and UVB radiation [29]. They possess melanin which provides them protection from UV radiation and is able to neutralize free radicals. In addition, melanin binds xenobiotics, thereby protecting cells from their harmful effects on the one hand, but on the other hand, leads to the accumulation of drugs in melanocytes and the possible enhancement of the cutaneous side effects of drugs [30,31]. The process of melanogenesis, which occurs in melanocytes, is connected with the generation of free radicals, causing melanin-producing cells to be exposed to relatively high levels of oxidative stress [32,33]. Melanin pigment can be found in two forms, eumelanin and pheomelanin, which differ in the impact on redox balance. Eumelanin exhibits antioxidant activity through the sequestration of iron ions and the direct interaction with oxidative radicals via electron transfer. In contrast, pheomelanin has pro-oxidant effects that are due to the generation of ROS during its photolysis. It is important to note that the synthesis of both types of pigment depends on the redox status as well as the availability of cysteine and glutathione in the reduced state. Therefore, the antioxidant status of the cell determines the quality of melanogenesis, i.e., whether the eumelanogenesis or pheomelanogenesis pathway will be more intense [34,35]. Taking into account UV radiation, in contrast to melanocytes, fibroblasts are only exposed to UVA radiation [29]. In the case of excess free radicals caused by UVA radiation and oxidants, fibroblasts can only rely on their antioxidant system. Fibroblasts have been shown to exhibit greater antioxidant enzyme activities compared to epidermal cells [36].

Under UVA radiation, FQs undergo photodegradation associated with the formation of free radicals, which are responsible for skin cell damage [14,15,16]. Since fibroblasts are exposed to UVA radiation they may also be affected by the phototoxic action of FQs induced by this range of radiation [29]. Taking this into account, we decided to analyze the phototoxic effects of LF on dermal fibroblasts. The studies also allowed us to compare the changes induced by UVA-activated lomefloxacin in pigmented and non-pigmented cells, as we had previously analyzed the response of melanocytes to LF. The comparison of the redox disturbance of melanin-containing and non-melanin-containing cells connected with the phototoxic action of LF is important because melanin significantly affects the oxidative stress level of cells. Since each type of melanin has a different effect on the redox state and melanogenesis itself disrupts the oxidative-reductive balance, the response of melanocytes to the action of an oxidizing agent such as UV-activated lomefloxacin may be different from that of fibroblasts, i.e., cells that do not possess and do not synthesize melanin [32,33]. In addition, it should be noted that in previous studies, we have shown that FQs also affect the process of melanogenesis, and this may also be related to the different susceptibility of fibroblasts and melanocytes to LF phototoxicity [20,37].

In these studies, cells were irradiated with UVA for 30 min at an intensity of 720 µW/cm^2^, corresponding to a dose of 1.3 J/cm^2^. It should be noted that The International Council for Harmonisation of Technical Requirements for Pharmaceuticals for Human Use hasn’t indicated standardized procedures for irradiation conditions in phototoxicity tests using in vitro assays [38]. The studies available in the literature for phototoxicity analysis have used different UVA doses, from 0.18 to 6.0 J/cm^2^ [39,40,41]. In the present study, fibroblasts were irradiated with the same dose of radiation that we used for melanocytes in the previous research. The applied radiation dose did not significantly affect the homeostasis of either fibroblasts or melanocytes, but induced the phototoxic activity of LF.

We have demonstrated that FQs alone, including LF, have cytotoxic effects on melanocytes with various degrees of pigmentation [18,19,37]. The results of current studies indicated that LF did not decrease fibroblast viability. We observed that LF caused only the inhibition of cell proliferation. LF-mediated alterations in fibroblast proliferation were also manifested in the cell cycle profile. We noted that LF increased the relative ratio of G1/S and G2-M/S, resulting primarily from a decrease in the number of cells in the S phase. The observed changes in the cell cycle led to an arrest in cell division and a decrease in the fibroblast number. It is known that FQs inhibit cell proliferation by affecting the cell cycle. Similar changes were observed in tendon fibroblasts and certain types of neoplastic cells (e.g., melanoma) exposed to FQs [42,43,44,45,46].

Our results showed that UVA radiation increased the cytotoxicity of LF to fibroblasts. Phototoxic action of the drug was observed for concentrations from 0.05 to 1.0 mM. In an in vitro study on melanocytes using the same protocol of drug and radiation treatment, we observed that LF had phototoxic effects in a broader range of concentrations, i.e., 0.001 to 1.0 mM [19]. The difference in the response of melanocytes and fibroblasts to the phototoxic action of LF may be due to the dissimilarity of these cells in their oxidoreduction status. Marrot et al. [47] demonstrated that normal human keratinocytes were also more susceptible to the phototoxic activity of LF than normal human fibroblasts.

We observed a significant reduction in cell viability in the case of exposure to UVA radiation and the drug at a concentration of 0.5 mM, thus we used this concentration of LF to assess changes in the cell redox system. The applied concentration of LF is higher than the concentration observed in a clinical trial when LF was administered *p.o*. [48]. It should be taken into account that LF binds to melanin, which may result in the accumulation of the drug in pigmented tissues and the achievement of a higher concentration of the drug in the skin than in the serum. Accumulation of LF in melanin-containing cells enhances its toxic effects, and cell damage may lead to drug release into the intercellular space and increase the exposure of surrounding cells to drug toxicity [31]. We have previously demonstrated that two classes of binding sites are involved in the LF–melanin complex formation. The calculated total number of binding sites was 0.92 µmol lomefloxacin per 1 mg melanin and was characterized by the following values of stability constants: K_1_ = 6.4 × 10^5^ M^−1^, and K_2_ = 7.03 × 10^2^ M^−1^ [18]. Furthermore, in previous studies, we analyzed changes in the redox system of melanocytes using LF at a concentration of 0.5 mM, among others, so we were able to compare differences in the response of melanocytes and fibroblasts to the phototoxic action of LF [19].

Non-enzymatic and enzymatic antioxidants i.e., glutathione, superoxide dismutase, catalase, and glutathione peroxidase are responsible for the maintenance of intracellular redox balance. SOD is an enzyme that catalyzes the dismutation reaction of superoxide anion radicals to oxygen and the less reactive hydrogen peroxide. There are two intracellular forms of this enzyme: cytosolic SOD1 with copper and zinc in the active center and mitochondrial SOD2 with manganese. CAT and GPx are responsible for hydrogen peroxide detoxification. CAT catalyzes the conversion of hydrogen peroxide to water and oxygen, and GPx reduces hydrogen peroxide to water at the expense of glutathione. GPx also utilizes GSH to remove lipid peroxides [27,49,50]. There are eight GPx isoforms, of which GPx1 exists commonly in the cytosol and mitochondria [51]. It is important to note that glutathione is involved in the removal of reactive oxygen species both through interactions with glutathione peroxidase and direct reaction with superoxide anion radicals [52]. Glutathione in its reduced form is a reservoir of intracellular reducing power that can be quickly used by cells in response to oxidative stress. By reducing the level of superoxide anion radicals and hydrogen peroxide, glutathione limits the generation of hydroxyl radicals in Fenton and Haber–Weiss reactions [50,51,53]. 

Oxidative stress is a result of not only increased free radical production but also diminished antioxidant defense. Therefore, the decrease in antioxidant levels attenuates processes that are responsible for preventing oxidative damage [54]. We observed that LF alone decreased the level of CAT and GPx as well as reduced glutathione in fibroblasts. The weakened removal of hydrogen peroxide may lead to an increase in the concentration of this type of ROS and the induction of oxidative modifications of cellular constituents [49,50]. FQs-mediated changes in antioxidant enzyme function were also demonstrated in other in vitro studies [55,56]. In the case of melanocytes, we showed that LF caused a different pattern of changes than in the fibroblasts [19]. LF increased the expression of hydrogen peroxide scavenging enzymes. Furthermore, the drug affected the levels of SOD1 and SOD2, an effect we did not observe in fibroblasts.

The obtained results indicated that UVA radiation enhanced LF-induced oxidative stress in fibroblasts. Cells exposed to LF and UVA exhibited the highest levels of ROS and the lowest level of reduced glutathione compared to other analyzed samples. It is known that under UVA irradiation, 8-halogenated FQs undergo dehalogenation to a singlet aryl cation, which reacts with molecular oxygen or water to form a superoxide radical and hydrogen peroxide [57]. The generated reactive oxygen species are responsible for oxidative damage to cell components [27]. Marrot et al. [47] analyzed oxidative DNA damage in skin cells exposed to UVA radiation and LF. It was observed that keratinocytes showed higher levels of oxidative DNA damage than fibroblasts.

It is notable that LF phototoxicity is associated with the induction of oxidative stress due to the increased production of ROS but also reduced capacity of the antioxidant system. The simultaneous exposure of fibroblasts to UVA radiation and LF caused significant changes in the expression of antioxidant enzymes. Previously, we demonstrated the same pattern of modifications in melanocytes [19]. UVA exposure of fibroblasts treated with LF resulted in decreased CAT levels with no increase in GPx expression. The observed changes may indicate a reduced ability to neutralize hydrogen peroxide. Moreover, irradiation of LF-exposed cells led to significant changes in the expression of SOD isoforms, a downregulation of cytosolic SOD1 and upregulation of mitochondrial SOD2. It should be noted that changes in the amount of SOD2 are moderate, and this may be relevant to the maintenance of mitochondrial function. Considering that the SOD2 isoform represents a much smaller fraction of the intracellular superoxide dismutase pool, we can hypothesize that the availability of the SOD1 isoform is critical for SOD function [58]. This is also consistent with our previous study showing that changes in superoxide dismutase activity were dependent on the amount of SOD1 [19]. Thus, decreased SOD1 expression may lead to reduced superoxide radical scavenging in cells exposed to UVA and LF. A high amount of superoxide radical and hydrogen peroxide is hazardous to cells because these two molecules may be transformed through iron-catalyzed Fenton and Haber–Weiss reactions to very reactive and harmful hydroxyl radicals. In order to determine the contribution of attenuated antioxidant systems to the pathomechanism of phototoxic action of LF, we evaluated whether the addition of an antioxidant would affect the oxidative stress induced by UV-activated LF. The obtained results revealed that incubation of cells with ascorbic acid reversed the oxidoreduction imbalance caused by simultaneous exposure to LF and UVA. Fibroblasts incubated with ascorbic acid after exposure to LF and UVA showed similar vitality associated with high levels of reduced glutathione to cells exposed to UVA only. Considering that the cells were incubated for only 24 h with ascorbic acid solution after exposure to the drug and UVA radiation, we hypothesize that the reversal of the drug effect by the addition of a small-molecule antioxidant was due to changes in glutathione oxidation. The phototoxic action of LF is associated with increased production of ROS, consequently leading to decreased levels of antioxidants in reduced form, such as glutathione, which are used to neutralize cell-damaging ROS.

## 5. Conclusions

Taken together, the presented results show that the phototoxic reaction induced by lomefloxacin may be connected with disturbances of homeostasis of the most numerous cells of the dermis, i.e., fibroblasts. It was observed that lomefloxacin exhibits antiproliferative effects on dermal fibroblasts by affecting their cell cycle. Lomefloxacin treatment was also associated with the decreased expression of hydrogen peroxide scavenging enzymes and the induction of oxidative stress. UVA radiation, as a trigger for the phototoxic activity of lomefloxacin, caused a cytotoxic effect of the drug on dermal fibroblasts. Moreover, it significantly potentiated the impact of the drug on cellular redox balance. Exposure to lomefloxacin and UVA radiation resulted in significant changes in intracellular antioxidant status and high ROS levels.

Analyzing the effects of lomefloxacin on dermal fibroblasts and melanocytes, it can be concluded that the phototoxic action of the drug is connected with the generation of a high level of oxidative stress, which results from both increased production of ROS and the weakened function of the antioxidant system. The oxidoreduction imbalance induced by UVA-activated lomefloxacin results in significant impairment of skin cell homeostasis.

## Data Availability

The data that support the findings of this study are available from the corresponding author upon reasonable request.
